# In Vivo Tumor Vascular Imaging with Light Emitting Diode-Based Photoacoustic Imaging System

**DOI:** 10.3390/s20164503

**Published:** 2020-08-12

**Authors:** Marvin Xavierselvan, Mithun Kuniyil Ajith Singh, Srivalleesha Mallidi

**Affiliations:** 1 Department of Biomedical Engineering, Tufts University, Medford, MA 02155, USA; marvin.xavierselvan@tufts.edu; 2 Research & Business Development Division, Cyberdyne INC, Cambridge Innovation Center, 3013 Rotterdam, The Netherlands; mithun_ajith@cyberdyne.jp; 3 Wellman Center for Photomedicine, Massachusetts General Hospital, Harvard Medical School, Boston, MA 02114, USA

**Keywords:** LED, photoacoustic imaging, ultrasound, tumor imaging

## Abstract

Photoacoustic (PA) imaging has shown tremendous promise for imaging tumor vasculature and its function at deeper penetration depths without the use of exogenous contrast agents. Traditional PA imaging systems employ expensive and bulky class IV lasers with low pulse repetition rate, due to which its availability for preclinical cancer research is hampered. In this study, we evaluated the capability of a Light-Emitting Diode (LED)-based PA and ultrasound (US) imaging system for monitoring heterogeneous microvasculature in tumors (up to 10 mm in depth) and quantitatively compared the PA images with gold standard histology images. We used a combination of a 7 MHz linear array US transducer and 850 nm excitation wavelength LED arrays to image blood vessels in a subcutaneous tumor model. After imaging, the tumors were sectioned and stained for endothelial cells to correlate with PA images across similar cross-sections. Analysis of 30 regions of interest in tumors from different mice showed a statistically significant R-value of 0.84 where the areas with high blood vessel density had high PA response while low blood vessel density regions had low PA response. Our results confirm that LED-based PA and US imaging can provide 2D and 3D images of tumor vasculature and the potential it has as a valuable tool for preclinical cancer research.

## 1. Introduction

The critical role of vasculature for tumor growth and metastasis is undisputed. Tumor aggressiveness and the extent of the aberrant tumor vascular structure and function are highly correlated. To meet the increasing needs for oxygen and nutrients, cancers induce neovascularization, one of the hallmarks of cancer [1]. The heterogeneity in tumor vasculature [2] affects drug distribution [3,4] and, as a result, impact treatment outcomes. Indeed, many anti-angiogenic agents or therapies that can prune these blood vessels and prevent nutrients from reaching the tumors have been developed [5]. Monitoring the dynamic changes in tumor vasculature post therapy is key for prognosis [6]. Several imaging modalities have been used to study changes in tumor vasculature both at structural and functional level. More recently, photoacoustic (PA) imaging has gained tremendous popularity for imaging tumor vasculature [7,8,9,10]. PA imaging involves detection of acoustic waves generated when the optical absorbers such as hemoglobin in the blood are irradiated with nanosecond pulsed light and undergo thermo-elastic expansion and contraction [11]. The acoustic waves are picked up by the ultrasound (US) transducer and processed to generate a PA image [11,12]. Notably, two features of PA imaging that make it highly suitable to image vasculature are: 1. ability to image blood vessels without exogenous contrast agents and 2. ability to complement US imaging that provides structural information of the tumor. 

Traditionally PA imaging systems use lasers (typically Nd: YAG pumped optical parametric oscillator lasers) for exciting the optical absorbers and employ a US transducer (array or single element) to receive the generated acoustic wave. These lasers are expensive, bulky with large spatial occupancy, and often have low repetition rate which leads to low image frame rate and acquisition speed. These limitations hinder the wide adaption of PA imaging systems in both preclinical and clinical applications. Advances in semiconductor industry have led to the creation of powerful Light-Emitting Diodes (LED) which are compact, portable, affordable, and energy-efficient. AcousticX by Cyberdyne Inc. (Tsukuba, Japan) is a LED-based system that can perform PA and US imaging at high frame rates. These LEDs can be fired with a pulse repetition frequency (PRF) from 1 to 4 kHz. Despite generating low optical output (400 µJ/pulse when using two 850 nm arrays), the high PRF of LEDs offers the possibility of averaging several frames which results in generating real-time PA images with reasonable signal-to-noise ratio (SNR) [13,14,15,16,17]. Furthermore, the spatial resolution and SNR provided by LED-based PA imaging is very similar and comparable to laser-based PA systems [13]. Due to the inherent advantages of the LED-based PA system, several research groups have explored its ability for various potential applications [15,16,18,19,20,21]. None of these studies provide validation of the generated PA image with histology. In this study, for the first time to the best of our knowledge, we report the LED-based PA imaging system’s ability to image microvasculature in subcutaneous murine tumor xenografts and validate the results using histology. 

## 2. Materials and Methods

### 2.1. LED-Based PA Imaging System

**Figure 1:**
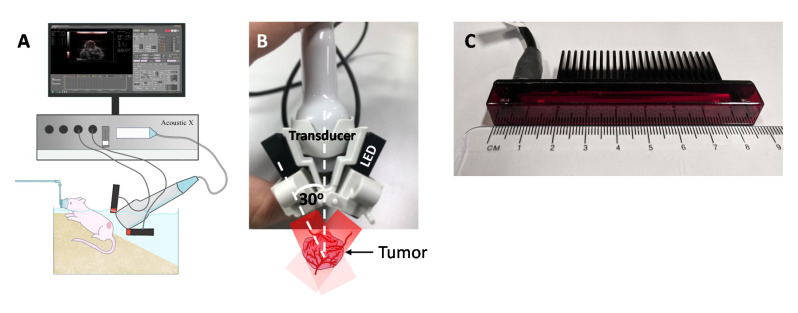
(**A**) Schematic of our experimental setup in which the Light-Emitting Diode (LED)-based photoacoustic (PA) system was utilized for imaging subcutaneous tumors in a mouse. (**B**,**C**) LED array used for illumination and the angle at which the LEDs are connected to the ultrasound transducer. (Scale bar = 2 mm).

An AcousticX system integrated with a 7 MHz US transducer (128 elements, −6 dB bandwidth is 80%) and two 850 nm LED arrays (for deeper light penetration) attached on either side of the transducer at a 30° angle was used for in vivo imaging (Figure 1A–C). The mean lateral and axial resolutions of AcousticX when using a 7 MHz transducer are 460 and 220 µm, respectively [14,19]. Each LED array had height, length and width dimensions of 12.4, 86.5, and 10.2 mm, respectively, and had 144 individual LEDs arranged in 4 rows (36 elements/row). The PRF of the LED array could be varied between 1, 2, 3, and 4 kHz. In this study, we operated the LED arrays at a PRF of 4 kHz to acquire and save PA images of the tumor vasculature at a frame rate of approximately 10 Hz i.e., 384 PA frames were continuously averaged to generate one display image. For offline MATLAB analysis, a higher number of frames (12,800) was averaged to enhance the PA image SNR. The SNR was defined as SNR = S/σ, where S is the mean of the image amplitude in a signal region, and σ is the standard deviation of the image amplitude in a noise region. The signal region was empirically chosen as a rectangular region (4 by 4 pixels) near a feeding blood vessel on the tumor surface where the PA signal was apparent; the noise region was manually chosen to correspond to a rectangular region outside the tumor region. 

### 2.2. Animal Model

All animal experiments were performed in compliance with the Institutional Animal Care and Use Committee (IACUC) of Massachusetts General Hospital (MGH). Swiss nu/nu female mice (aged 6–8 weeks, body weight 20–25 g) were raised in aseptic conditions in the institution’s animal facility with a 12 h light and dark cycles. We used human hypopharyngeal squamous cell carcinoma (FaDu) xenograft model because these are highly vascularized tumors [22,23]. FaDu cells were cultured in Eagle’s Minimum Essential Medium supplemented with 10% fetal bovine serum and 1% antibiotics (Penicillin and Streptomycin 1:1 *v*/*v*) in a T75 flask and maintained at 37 ℃ and 5% CO_2_. At 80–90% confluency, cells were trypsinized and centrifuged to remove the supernatant. The cells were then suspended in a mixture of matrigel (BD Bioscience, CA) and phosphate buffered saline in 1:1 *v*/*v* at a density of 1 million cells per 100 µL and injected subcutaneously into mice. 

### 2.3. In-Vivo Photoacoustic Imaging

A total of 3 mice were imaged using the AcousticX system when the tumors reached 6–8 mm in diameter as measured with a digital caliper (VWR 62379-531, Radnor, PA, USA). The mice were anesthetized using 2% isoflurane and placed in a warm water bath to achieve optical coupling with the US transducer as shown in Figure 1A. The LED arrays were positioned at a 30° angle (Figure 1B) to the US transducer to colocalize the light with the transducer focus. The US transducer was then vertically positioned such that the tumor was approximately at the focal region of the transducer (~20 mm depth). Radio frequency (RF) data and US/PA images for several cross-sections of the tumors were acquired and saved for further analysis. Motion artifacts in the tumor region due to mouse breathing were minimal as shown in Video S1 (captured at 10 Hz frame rate, see Supplementary materials). In the post-acquisition image analysis, frames with breathing motion were not included. The RF data was processed with previously reported algorithms [24,25,26,27] using custom written MATLAB code to obtain US/PA images of tumor vasculature. Three-dimensional imaging was performed by moving the US/PA transducer linearly across the XY plane with the aid of an in-built translation stage and the sequence was rendered in ImageJ.

### 2.4. Histology

Post imaging mice were sacrificed, and tumors were surgically removed without skin. The tumors were embedded and frozen in optical cutting temperature (OCT) compound in the same orientation as the US/PA image with the aid of fiducial markers placed prior to euthanasia. The frozen tumors were cryosectioned (5 µm) in the same plane as the US/PA images based on previously published methodology [28] and stained for platelet endothelial cell adhesion molecule-1 (or CD31). Tissue sections were incubated with anti-CD31 primary antibody (1:50 dilution; Rabbit polyclonal anti-CD31, Abcam) overnight at 4 ℃, washed, and incubated with fluorescently tagged secondary antibody (1:100 dilution, Donkey Anti-Rabbit IgG NL637 Affinity Purified Ab, R&D systems) for one hour. The sections were mounted using slowfade gold antifade mountant with 4′,6-diamidino-2-phenylindole (DAPI, Invitrogen, Carlsbad, CA, USA) and sealed with coverslips. A whole-slide scanning fluorescence imaging system (Hamamatsu NanoZoomer 2.0-HT) was used to image the slides at 40 x magnification and images were saved in Hamamatsu Nanozoomer Digital Pathology Image (NDPI) format. 

### 2.5. Image Processing

**Figure 2:**
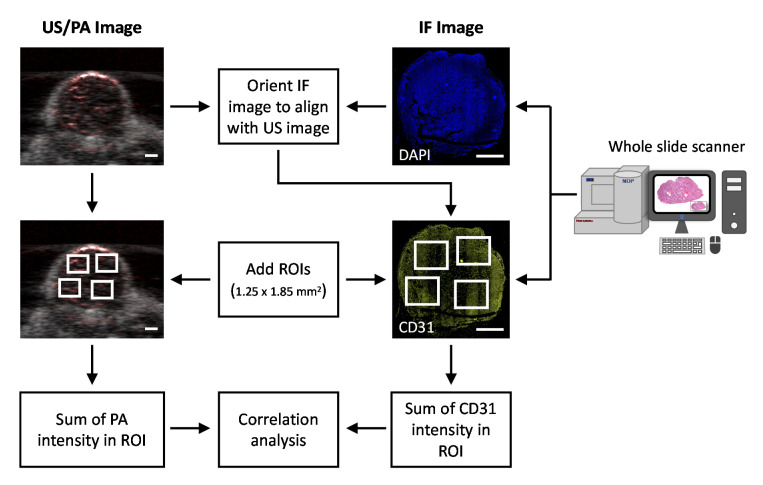
Flowchart describing the image processing methodology to correlate CD31 signal intensity with PA signals. (Scale bar = 2 mm).

A 5x magnification of the fluorescence image was extracted from the NDPI file using ImageJ, rotated to match the PA image orientation and saved for further analysis in MATLAB (Figure 2). Several 1.25 × 1.85 mm^2^ regions of interest (ROI) were chosen from the immunofluorescence (IF) and PA images for correlation analysis. This corresponds to 679 × 1005 and 5 × 50 pixels in IF and PA images, respectively. The background from a similar ROI area was calculated from a region outside the tumor area for both IF and PA images and subtracted from the tumor ROI values. The CD31 intensity from the ROI in IF images were summed and compared to the total PA signal intensity from the corresponding ROI in the PA image. Linear regression analysis was performed using GraphPad Prism software.

## 3. Results

### 3.1. SNR Enhances with Frame Averaging

**Figure 3:**
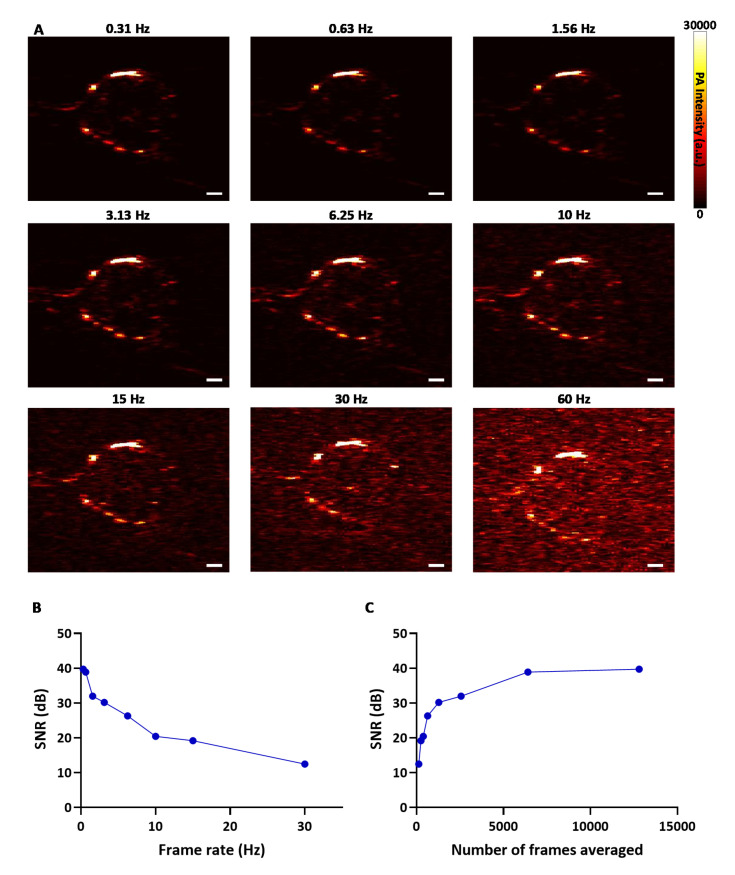
The signal-to-noise ratio (SNR) is enhanced by averaging several frames in the LED-based PA imaging system. (**A**) PA images of a tumor acquired and processed at different frame rates. (**B**) Relationship between the frame rate and SNR. (**C**) Plot detailing the SNR change with respect to the averaging of frames. (Scale bar = 2 mm).

The PA image of a representative tumor acquired and processed at different frame rates (0.31–60 Hz) is shown in Figure 3A. To enhance the SNR and improve image quality, subsequent PA frames were averaged resulting in lower frame rate. This relationship between the frame rate and SNR of the PA image is displayed in Figure 3B, where low frame rate offers better SNR as expected. The low SNR of the image at higher frame rates may be attributed to the low output power of the LED, however, due to the high pulse repetition rate of LED excitation for PA data acquisition, more PA frames can be acquired and averaged to increase the SNR (Figure 3C). A frame rate of 10 Hz was chosen for data acquisition (2D and 3D acquisition) during the experiment as it provided the best possible SNR for near real-time combined US/PA imaging. To quantitatively compare the 2D images with IF images, the acquired PA images were further averaged to a final frame rate of 0.31 Hz to enhance the SNR by 20 dB, i.e., overall 12,800 frames were averaged to obtain an SNR of ~40 dB (Figure 3C). The enhanced SNR facilitated accurate comparison with histology images without the involvement of background noise. 

### 3.2. Tumor Vascular Imaging with AcousticX

**Figure 4:**
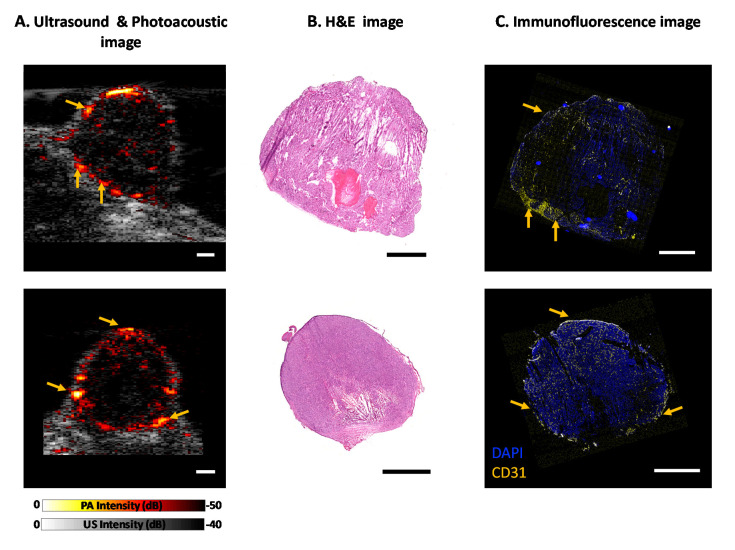
Evaluation of AcousticX on subcutaneous tumor xenografts in mice. (**A**) Ultrasound and photoacoustic image overlay of the tumor vasculature of two mice. Images of the Mouse 1 tumor acquired at a frame rate of 10 Hz are shown in Video 1. Motion due to breathing was minimal and only frames that did not have motion artifacts were used for image analysis. (**B**) H&E stain of the tumor cross-section. (**C**) Immunofluorescence stain for blood vessels in yellow and cell nuclei in blue. (Scale bar on all images = 2 mm, Video S1, MP4, 1.7 MB).

Figure 4A shows the overlay of the US image (in grayscale) with the PA image of two representative tumors (offline reconstructed). The B-mode US image depicts the shape and structure of the tumor (~6 to 10 mm in diameter and depth), and the PA image provides the blood vessels location inside and around the tumor. The PA image is displayed on a pseudo-colored hot map where low and high signals are represented by black and white, respectively. Subcutaneous tumors are fed by large blood vessels usually originating from the skin tissue above or the muscle tissue lying beneath the tumor, as visualized in Figure 4A. AcousticX can detect the PA signal not only from the surface of the tumor but also from the deep edge (~10 mm) of the tumor. The Hematoxylin and Eosin (H&E) stain is a standard histology stain to showcase tissue microanatomy. Figure 4B showcases the H&E image of the tumor at the same cross-section as the US/PA image where the tumor structure is analogous to the shape and size observed in the US image. Figure 4C shows the IF image of the tumor cross-section with CD31 stain for blood vessels (yellow) and DAPI for cell nuclei (blue). DAPI staining was performed to visualize the tumor structure and aided in selecting corresponding ROIs as the US/PA image. Qualitatively we noticed regions of the high PA signal have high vessel density on the IF images as displayed by the yellow arrows in Figure 4. The blood vessels on the top of the tumor (particularly Figure 4A top panel) are not strongly visualized in the IF image as these vessels were part of the skin above the tumors which was removed during the histological processing of the tissue. 

### 3.3. Correlation of PA Signal with Histology

**Figure 5:**
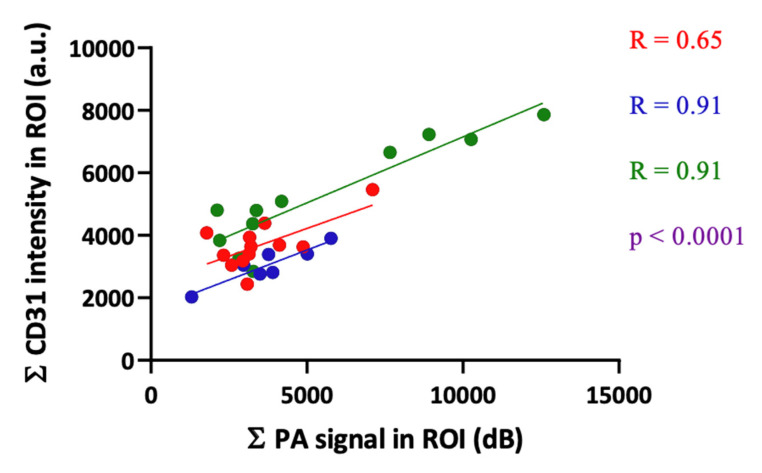
Correlation between background subtracted signal intensity from PA images and CD31 immunofluorescence (IF) image. Each color represents data from different mice. A total of 30 ROIs from three mice were used in our analysis. The R-value for correlation based on Pearson’s coefficient was calculated between the CD31 intensity from the IF image and PA signals from blood vessels.

The correlation plot between the PA signal from blood vessels and CD31 intensity from the IF image is displayed in Figure 5. As expected, we observed that larger blood vessels or areas with high blood vessel density had a high PA signal while the micro vessels or low blood vessel density regions had a low signal. The overall R-value for correlation was 0.84 and individual R-values for the three tumors used in the study were 0.65, 0.91 and 0.91. The differences in the R-values among the tumors were due to the inter-tumor heterogeneity in the vasculature and biological variations across different animals. The results obtained here with the LED-based PA imaging system are on par with reports using traditional laser-based PA imaging systems. For example, Mallidi et al. [28] and Bar-Zion et al. [29] qualitatively demonstrated good spatial co-registration between tumor vascular oxygenation maps obtained from PA imaging and hypoxia markers such as pimonidazole or carbonic anhydrase IX. Gerling et al. [30] observed an excellent R-value of 0.95 when quantitatively correlating oxygenation saturation measurements from PA imaging to the intensity of pimonidazole in the tumor, however, there were only six points in this correlation. Furthermore, the PA signals were averaged over the entire tumor underplaying the intra-tumor heterogeneity. In our study we divided the tumor region into several ROIs to consider both high and low vascular regions. In addition, these studies [28,29,30] utilized higher frequency transducers with limitations in imaging depth. It is obvious that some microvascular structures inside the tumors may be not within the detection limits of the 7 MHz transducer used in our study, which would have resulted in slightly lower correlation between PA signals and CD31 intensity in certain regions of the tumor. Nevertheless, we clearly demonstrate a good correlation between histology and PA images generated using an LED source with comparatively less optical output energy.

### 3.4. 3D Tumor Imaging with AcousticX

**Figure 6:**
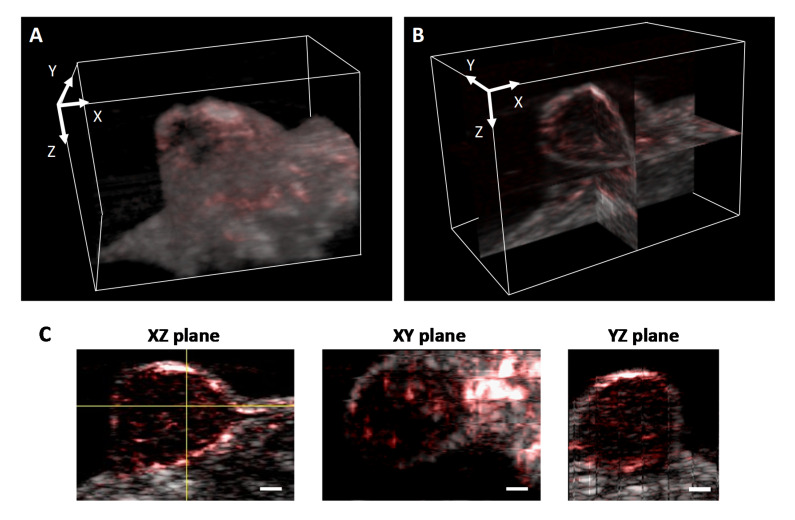
(**A**) Overlay of ultrasound and photoacoustic images of tumors reconstructed in 3D (Video 2) and (**B**) in orthoslice view. (**C**) Tumor images displayed in different 2D planes. (Scale bar = 2 mm, Video S2, MOV, 779 kB).

An LED-based photoacoustic imaging system can also be used to visualize vasculature in 3D. Figure 6 shows the overlay of a US/PA image of tumor acquired by AcousticX at a frame rate of 10 Hz and reconstructed in 3D using ImageJ. A corresponding video showcasing the 3D rendering of US/PA images of the tumor is shown in Video S2. Figure 6C shows the structure of the tumor in different cross-sectional planes. The measurements obtained from AcousticX images have good agreement with the caliper measurements for the tumor size (length, width, and depth). Two LED arrays on either side of the US transducer delivered a total optical energy output of 400 µJ/pulse on the skin surface. In the absence of optical scattering, the irradiation area at the US focus will be approximately 50 × 7 mm in the YX-plane, with a maximum radiant exposure of 0.11 mJ/cm^2^, which is orders of magnitude lower than the ANSI safety limit and the radiant exposure in laser-based PA imaging systems. It is encouraging that the system used in this study could visualize tumor vasculature in vivo at an imaging depth of 10 mm, considering the low pulse energies provided by the LED arrays.

## 4. Discussion

In preclinical cancer research, rodent models are widely used to study the complex physiological process involved in tumor formation and therapy response. The tumor is grown either subcutaneously or orthotopically at the site of the origin of tumor cells. The maximum size for subcutaneous tumors is less than 1–2 cm in diameter, and orthotopic tumors depending on the organ can vary between 3 and 6 mm in diameter. The length of the LED array in AcousticX is 8.65 cm [14] and is significantly larger than that of the tumors generally seen in preclinical research. Hence most of the light from the LED arrays is delivered outside of the tumor. Considering this, the imaging depth of ~10 mm we achieved demonstrated the high sensitivity of the system, which is commendable. Better light delivery strategies to illuminate tumors efficiently may help in improving the imaging depth and SNR further. 

Currently the AcousticX system is integrated with a 7 MHz ultrasound transducer that has limitations in spatial resolution. Higher frequency transducers offer better resolution and our future work involves integrating these transducers with LED arrays to enhance image resolution. In addition to improvements in spatial resolution, image SNR can be enhanced through novel beamforming algorithms. Traditional beamforming algorithms are based on “delay and sum” and “delay-multiply and sum” methodology. Although they are simple to implement in imaging systems, they generate low quality images with low contrast, especially when the data is noisy. Recent studies have shown that the “double stage delay-multiply and sum” algorithm improves lateral resolution and offers high contrast for an LED-based PA imaging system [31,32]. Moreover, in this study we used a single wavelength (850 nm) LED array that provides deeper light penetration to image tumor vasculature. The availability of high-power multi-wavelength LED arrays will enable imaging blood oxygen saturation levels in the tumor vasculature [33,34]. This information is key for monitoring treatment response and predicting recurrence. These multi-wavelength LED arrays can also enable the imaging of tumor via receptor targeted exogenous contrast agents [15,18,25,27], as previously demonstrated by us and others with traditional pulsed laser sources [9,35,36,37,38,39,40,41]. With the availability of low-cost multi-wavelength PA imaging systems, our future studies will aim at developing and monitoring various targeted therapies guided by multi-wavelength PA imaging that can provide vascular information with information on the localization of targeted contrast agents.

## 5. Conclusions

In summary, we demonstrate the capability of an LED-based PA imaging system for monitoring tumor vasculature in vivo. The PA images obtained are in good correlation with the gold standard IF images. With the widespread rise of PA imaging technology, new improved reconstruction algorithms, high frequency transducers, better illumination strategies, and multi-wavelength LED arrays, we can expect LED-based PA imaging to become a promising tool and play a highly significant role in both preclinical research and clinical applications.

## Supplementary Materials

The following are available online at https://www.mdpi.com/1424-8220/20/16/4503/s1, Video S1: Visualization demonstrates that the motion artifacts in the tumor region caused due to mouse breathing, while acquiring the RF data for Figure 4 in the manuscript, is minimal. Video S2: Visualization demonstrating the 3D rendering of US/PA images of the tumor acquired by AcousticX.
